# HLA class I NK-epitopes and KIR diversities in patients with multiple myeloma

**DOI:** 10.1007/s00251-024-01336-w

**Published:** 2024-03-13

**Authors:** Nicky A. Beelen, Stefan J. J. Molenbroeck, Lisette Groeneveld, Christien E. Voorter, Gerard M. J. Bos, Lotte Wieten

**Affiliations:** 1 Division of Hematology, Department of Internal Medicine, Maastricht University Medical Center+, Maastricht, the Netherlands; 2 GROW-School for Oncology and Reproduction, Maastricht University, Maastricht, the Netherlands; 3 Department of Transplantation Immunology, Tissue Typing Laboratory, Maastricht University Medical Center+, Maastricht, the Netherlands

**Keywords:** Natural killer cells, Human leukocyte antigen genes, Killer-cell immunoglobulin-like receptor, Multiple myeloma, Association

## Abstract

Multiple myeloma (MM) is a hematological malignancy caused by the clonal expansion of malignant plasma cells in the bone marrow. Myeloma cells are susceptible to killing by natural killer (NK) cells, but NK cells fail to control disease progression, suggesting immunosuppression. The activation threshold of NK-effector function is regulated by interaction between KIRs and self-HLA class I, during a process called “education” to ensure self-tolerance. NK cells can respond to diseased cells based on the absence of HLA class I expression (“Missing-self” hypothesis). The HLA and KIR repertoire is extremely diverse; thus, the present study aimed to characterize potential variances in genotypic composition of HLA Class I NK-epitopes and KIRs between MM patients and healthy controls. Genotypic expression of KIR and HLA (HLA-C group-C1/C2 and Bw4 motifs (including HLA-A*23, A*24, A*32) were analyzed in 172 MM patients and 195 healthy controls. Compared to healthy controls, we did not observe specific KIR genes or genotypes, or HLA NK-epitopes with higher prevalence among MM patients. The presence of all three HLA NK-epitopes (C1^+^C2^+^Bw4^+^) was not associated with MM occurrence. However, MM patients were more likely to be C1^-^/C2^+^/Bw4^+^ (*p* = 0.049, OR 1.996). In line with this, there was a trend of increased genetic co-occurrence of Bw4 and KIR3DL1 in MM patients (*p* = 0.05, OR 1.557). Furthermore, MM patients were more likely to genetically express both C2/KIR2DL1 and Bw4/KIR3DL1 (*p* = 0.019, OR 2.453). Our results reveal an HLA NK-epitope combination that is associated with the occurrence of MM. No specific KIR genotypes were associated with MM.

## Introduction

Multiple myeloma (MM) is a hematological malignancy caused by the clonal expansion of malignant plasma cells in the bone marrow (BM), leading to the presence of monoclonal M-proteins and/or the light chain part of the proteins in the blood or urine and subsequent organ dysfunction (Kumar et al. 2017). Natural killer (NK) cells, as part of the innate immune system, play a crucial role in the recognition of diseased cells by selectively releasing toxic granules and by inducing target cell death (Wu and Lanier 2003). While several *in vitro* and *in vivo* studies have demonstrated the involvement of NK cells in controlling MM (Clara and Childs 2022; Godfrey and Benson Jr 2012; Guillerey et al. 2015; Ponzetta et al. 2015), there is evidence suggesting that MM exhibit resistance to NK cell-mediated killing, necessitating additional stimulatory triggers for NK cell activation (Carlsten et al. 2019; Mahaweni et al. 2018). Consequently, the failure of NK cells to control disease progression might indicate the presence of immunosuppression in the MM setting (Godfrey and Benson Jr 2012). Notably, the high expression of NK cell inhibitory molecules on MM cells has been identified as a factor impeding the cytotoxic potential of NK cells (Carbone et al. 2005; Sarkar et al. 2015).

The activation of NK cell cytotoxicity is tightly controlled by the balance between signaling induced by their activating and inhibitory receptors. Key inhibitory receptors, including inhibitory killer immunoglobulin-like receptors (iKIR) and natural killer group 2 member A (NKG2A), specifically recognize the human leukocyte antigens (HLA) (Pende et al. 2019). The interaction between KIR or NKG2A, and their respective self-HLA ligands induces NK cell functional competence through a process known as “education,” resulting in the acquisition of the cytotoxic effector molecules, altered metabolism, and enhanced effector function (Goodridge et al. 2019; Kim et al. 2005). Education ensures self-tolerance and, according to the “missing self-hypothesis,” renders NK cells able to recognize diseased cells based on the absence of HLA class I expression (Ljunggren and Kärre 1990).

KIRs are encoded within the leucocyte receptor complex on chromosome 19 and segregate independently from HLA genes on chromosome 6 (Kärre 2002). Therefore, it is possible for an individual to lack the specific KIR required for the corresponding self-HLA Class I ligand, resulting in the NK cell being uneducated for that particular KIR-HLA ligand pair. Due to stochastic expression of KIRs, there is also a possibility that NK cells may not express any KIR for the self-HLA, leading to the presence of uneducated NK cells that display hyporesponsiveness. Uneducated NK cells require heightened levels of activation to initiate a response. Consequently, NK cell education plays a pivotal role in regulating the threshold of NK cell effector function (Anfossi et al. 2006; He and Tian 2017).

In terms of specificity, KIRs recognize certain HLA alleles (HLA NK-epitopes): KIR2DL1 recognizes the HLA-C group-C2 epitope, defined by Asn77 and Lys80, while KIR2DL2/3 recognizes the HLA-C group-C1 epitope, defined by Ser77 and Asn80. Two HLA-B allele groups (HLA-B*46 and HLA-B*73) classify as a C1 motif, and B*46:01 and B*73:01 were shown to be recognized by KIR2DL2/3 (Moesta et al. 2008). KIR3DL1 recognizes HLA-B molecules carrying a Bw4 motif and HLA-A*23, A*24, and A*32 (Kärre 2002). Certain HLA-A alleles, HLA-A*03 and HLA-A*11, can be recognized by KIR3DL2 (Hansasuta et al. 2004). In addition, the non-classical HLA-F and HLA-G are ligands for KIR3DL2 and KIR2DL4, respectively. The independent segregation of KIR and HLA genes allows for a multitude of possible combinations of KIR and HLA pairs (Pende et al. 2019). Additional KIRs can be expressed by NK cells. In fact, 14 KIR genes (KIR2DL1, -2DL2, -2DL3, -2DL4, -2DL5, -2DS1, -2DS2, -2DS3, -2DS4, -2DS5, -3DL1, -3DS1, -3DL2, -3DL3) and 2 pseudogenes (-2DP1 and -3DP1) have been described (Campbell and Purdy 2011; Pende et al. 2019). The combination of KIR-genes can be further classified into the A haplotype, which includes only a single activating KIR, and the B haplotypes, which contains multiple activating KIRs (Pende et al. 2019).

The ability of educated NK cells to detect whether a target lacks self-HLA class I expression, thereby meeting the criteria of “missing-self” (Anfossi et al. 2006; Long et al. 2013), can be exploited to reduce the activation threshold of NK cells in the setting of KIR-ligand mismatched haploidentical transplantation or infusion of haploidentical donor NK cells (Ruggeri et al. 2016). Here, donor NK cells, expressing KIR(s) but without corresponding recipient HLA class-I ligands, can detect the missing self-class I ligand, leading to beneficial anti-tumor alloreactivity. Typically, only 30% of the population is genetically positive for all three HLA NK-epitopes (Ruggeri et al. 2016). When examining the HLA typing of an initial cohort of MM patients in our medical center, we observed that 80% (8 out of 10) of patients expressed all three HLA NK-epitopes, HLA-C C1, -C2, and the Bw4 motif (Mahaweni et al. 2018). This observation would render these patients ineligible for a KIR-ligand mismatched transplantation. However, since this was an initial study with a very small patient cohort, we set out to study whether this trend holds true in a larger patient cohort. Therefore, the objectives of this study were to validate this observation and, in addition, to identify potential differences in the distribution of HLA-class I NK-epitopes and combinations thereof between patients with MM and a healthy cohort. Additionally, we assessed whether specific KIR genes or KIR genotypes were associated with MM. Furthermore, we characterized potential variances in the distribution of KIR-HLA ligand pairs, which mediate NK cell education, between patients with MM and healthy controls.

## Materials and methods

### Study population and ethics statement

The study population consisted of 172 patients with MM who underwent stem cell transplantation between 2005 and 2012. The control group comprised of 195 healthy blood bank donors that were registered between 2012 and 2013. Missing cases for total KIR genotyping are as follows: 1 in patients (identification KIR2DS3 failed), 1 in healthy controls (identification KIR2DS2 failed). The study was performed in agreement with “Non WMO” the “Code for Proper Secondary Use of Human Tissue in the Netherlands” and was approved by the local ethics committee (METC 2019-1381).

### DNA extraction and KIR and HLA genotyping

Genomic DNA was extracted from ethylenediamine tetraacetic acid (EDTA) blood samples using the QIAamp DNA blood mini kit (Qiagen, Cat. No. 51106). The presence of HLA epitopes known to interact with KIR on NK cells (HLA Bw4 motifs (including HLA-A*23, A*24, A*32, not A*25), HLA-C C1 epitope, and HLA-C C2 epitope) was determined using Luminex^®^ sequence-specific oligonucleotides (SSO) analysis (One Lambda, Thermofisher) according to manufacturer’s instructions. When low-resolution typing (SSO) did not provide definitive results, we conducted high-resolution typing to achieve a conclusive typing. Of the 172 patients, 37 were typed at high resolution. Of the 195 healthy controls, 49 were typed at high resolution. High-resolution typing by long-read Nanopore sequencing was done for HLA class I genes as described by Matern et al. or high-resolution typing by Sanger sequence-based typing (SSBT) for HLA class I genes as described by Voorter et al. (Matern et al. 2020; Voorter et al. 2014). The presence of the different KIR genes was determined using the KIR SSO Genotyping Test (RSSOKIR, One Lambda, Thermo Fisher) according to the manufacturer’s instructions in conjunction with Luminex FlexMap 3D with xPotent 4.2 and analyzed using HLA Fusion 6.1.0 software.

### Statistical analysis

Frequencies of the HLA NK-epitopes, KIR genes, KIR AA and Bx genotypes, and NK cell education potential were compared between MM and healthy references. Potential associations between the presence of MM and the independent variables were estimated by Pearson chi-square or two-tailed Fisher’s exact test. Significant differences (*p* < 0.05) were assessed by odds ratio. All analyses were done with SPSS Version 27 (IBM SPSS statistics).

## Results

### HLA NK-epitope profile distribution among MM patients and healthy controls

We first analyzed the genetic presence of HLA NK-epitopes, specifically HLA-C C1 and HLA-C C2 groups, and HLA-Bw4 motifs, which are recognized by KIRs expressed on NK cells. None of the patients or healthy controls showed the presence of HLA-B*46 or B*73. We did not observe significant differences between the MM patients and healthy control cohorts for the genotypic presence of specific HLA NK-epitopes (Table 1). Next, we examined whether specific combinations of these HLA NK-epitopes exhibited a higher genetic prevalence in patients with multiple myeloma (MM) compared to healthy controls. MM patients were more likely to have the C1^−^/C2^+^/Bw4^+^ HLA genotype (7.2% in controls vs 13.2% in MM patients, *p* = 0.049, OR 1.996). We did not observe a significant association between the presence of all three HLA NK NK-epitopes and occurrence of MM. Of the patients, 32.0% was positive for all three HLA NK-epitopes, which was 36.4% in healthy controls.

**Table 1:** HLA NK-epitope frequencies among patients with MM and healthy controls

	**Control (*n* = 195)**	**Patient (*n* = 172)**		
	*%*	*%*	*p-value*	*OR (CI)*
*C1 positive*	91.3	85.5	0.081	
*C2 positive*	56.4	56.4	0.998	
*Bw4 positive*	66.7	73.8	0.135	
*C1*^*+*^*/C2*^*−*^*/Bw4*^*−*^	20.5	15.1	0.179	
*C1*^*−*^*/C2*^*+*^*/Bw4*^*−*^	1.5	1.2	1.000^A^	
*C1*^*+*^*/C2*^*+*^*/Bw4*^*−*^	11.3	9.9	0.664	
*C1*^*+*^*/C2*^*−*^*/Bw4*^*+*^	23.1	28.5	0.236	
*C1*^*−*^*/C2*^*+*^*/Bw4*^*+*^	7.2	13.4	0.049	1.996 (0.992–4.014)
*C1*^*+*^*/C2*^*+*^*/Bw4*^*+*^	36.4	32.0	0.372	

*OR* odds ratio, *CI* confidence interval. *p* < 0.05: statistically significant; based on two-tailed Pearson chi-square test or ^A^Fishers’s exact test (two-sided)

### No specific KIR genes or genotypes with increased prevalence in MM patients

The distribution of 16 KIR genes was determined in patients and healthy controls. Taken together, 27 different KIR genotypes were observed in the study population and genotype 1, 4, and 2 were the three most frequently occurring genotypes in both populations (Table 2). As expected, framework genes (3DL3, 3DP1, 2DL4, 3DL2) were present in all subjects (Table 2). Next, we evaluated a potential association between individual KIR genes and occurrence of MM. We did not observe specific KIR genes with higher prevalence among MM patients compared to healthy controls (Table 3).

**Table 2:**
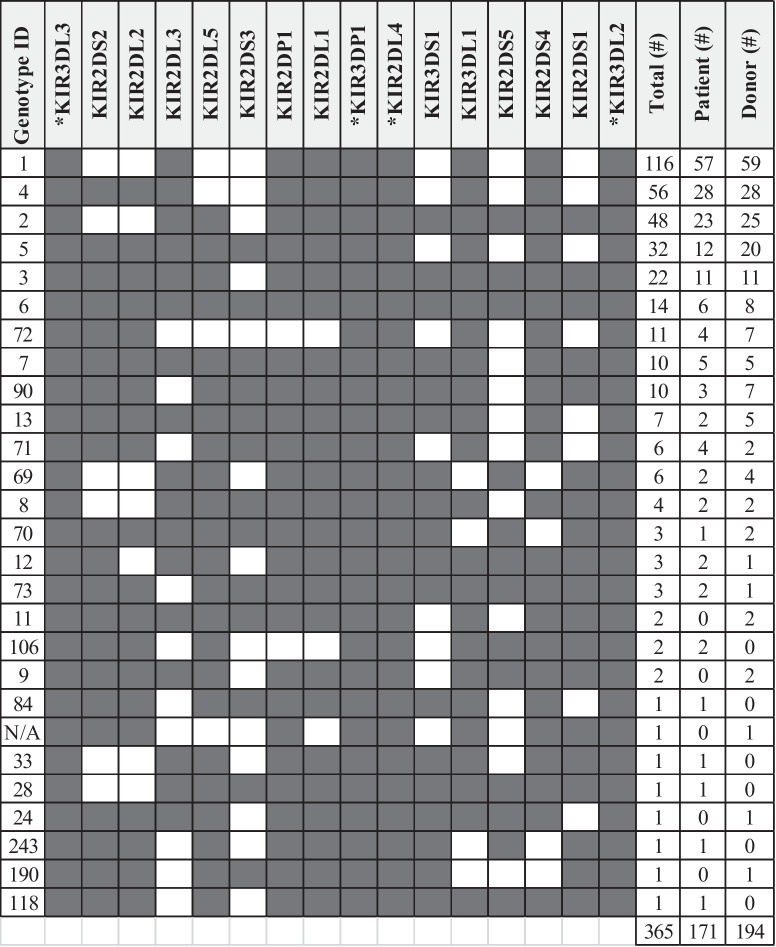
KIR genotypic profile distribution among study population

Overview of the observed KIR genotypes and distribution among MM patients (*n* = 171) and healthy controls (*n* = 194), sorted for the total number. Missing cases for total KIR genotyping: 1 in patients (identification KIR2DS3 failed), 1 in healthy controls (identification KIR2DS2 failed). *Indicates KIR framework gene. Note: Genotype ID was obtained from the online database http://www.allelefrequencies.net. N/A was not yet registered in the respective database

**Table 3:** KIR genes among MM patients and healthy controls

	**Control (*n* = 194)**		**MM patients (*n* = 171)**		
	*n*	*%*	*n*	*%*	*p-value*
*2DL1*	187	95.9	166	96.5	0.759
*2DL2*	103	52.8	84	48.8	0.446
*2DL3*	176	90.3	154	89.5	0.819
*2DL5*	100	51.3	82	47.7	0.490
*3DL1*	187	95.9	168	97.7	0.339
*2DS1*	73	37.4	63	36.6	0.873
*2DS2*	104	53.6	86	50.0	0.490
*2DS3*	55	28.2	39	22.7	0.238
*2DS4*	187	95.9	168	97.7	0.339
*2DS5*	56	28.7	52	30.2	0.751
*3DS1*	74	38.0	64	37.2	0.884
*2DP1*	188	96.4	166	96.5	0.958

Comparison of KIR gene distribution for MM patients (*n* = 171) compared to healthy controls (*n* = 192). Missing cases for total KIR genotyping: 1 in patients (identification KIR2DS3 failed), 1 in healthy controls (identification KIR2DS2 failed). A *p*-value of <0.05 was considered significant based on two-tailed Pearson chi-square test. Framework genes KIR2DL4, KIR3DL2, KIR3DL3, and KIR3DP1 were present in all individuals

Due to the strong linkage disequilibrium (LD) observed among KIR genes, it is difficult to determine the individual impact of each KIR on NK cell response (Hsu 2002a, b). However, based on the presence or absence of specific KIR genes, individuals can be classified into distinct genotypes. The A genotype (AA) (Table 2; Genotype ID 1), represents a fixed combination of genes, characterized by the presence of only one activating KIR (aKIR), i.e., KIR2DS4, while all other combinations are collectively referred to as B genotypes (Bx) (Hsu et al. 2002a, b). The AA and Bx genotype frequencies in MM patients were 33.3% and 66.7%, respectively (Table 4). Within healthy controls, the AA and Bx genotype frequencies were 30.4% and 69.6%, respectively. We did not observe an association between the presence of either an AA or Bx genotype and presence of MM disease (*p* = 0.550). The KIR2DS4 gene can be divided into two distinct versions: a full-length sequence and the other featuring a 22-nucleotide deletion. This deletion leads to a frameshift mutation, yielding a truncated protein that fails to be expressed on the cell surface of NK cells (Hsu et al. 2002a, b). The occurrence of both full-length and truncated KIR2DS4 alleles showed comparable frequencies between patients and healthy controls. Furthermore, the prevalence of a KIR A haplotype lacking any KIR-mediated activating potential, as denoted by the presence of the KIR2DS4^del/del^ allotype, was comparable between patients and healthy controls (Table 4).

**Table 4:** KIR AA vs Bx genotype distribution among study population

	**Control (*n* = 195)**	**Patient (*n* = 172)**	
	*%*	*%*	*p-value*
*AA vs Bx* *(%AA*^*+*^ *shown)*	30.4	33.3	0.550
Centromeric			0.677^A^
*A/A*	46.4	50.3	
*A/B*	43.8	39.2	
*B/B*	9.8	10.5	
Telomeric			0.779^A^
*A/A *	59.8	61.4	
*A/B *	36.6	36.3	
*B/B *	3.6	2.3	
B content score			0.949^A^
*0 *	30.4	33.3	
*1 *	40.7	40.4	
*2 *	22.7	19.9	
*3 *	5.7	5.9	
*4 *	0.5	0.6	

	**Control (*n* = 187)**	**Patient (*n* = 168)**	
	*%*	*%*	*p-value*
***KIR2DS4*** alleles and allotypes			
*2DS4*^*del*^	81.8	82.1	0.937
*2DS4*^*full*^	41.7	47.5	0.263
*2DS4*^*full/full*^	18.2	17.9	0.973
*2DS4*^*full/del*^	23.5	29.8	0.184
*2DS4*^*del/del*^	58.3	52.4	0.263

	**Control (*n* = 57)**	**Patient (*n* = 59)**	
	*%*	*%*	*p-value*
***KIR A*** haplotype and ***2DS4***^***del/del***^	64.4	57.9	0.472

KIR AA vs Bx genotypes, distribution of centromeric or telomeric regions, and KIR B-content score were based on the inhibitory or activating KIR gene content, and their frequencies among the cohorts (as described by Cooley et al. 2010) based on the content of the inhibitory or activating KIR genes, and their frequencies among the cohorts. The presence of KIR2DS4 full-length alleles or those with a 22-nucleotide deletion, as well as the characterization of KIR2DS4 allotypes, is determined within the cohorts excluding KIR2DS4-negative individuals. The calculation of the KIR A haplotype carrying the KIR2DS4^del/del^ allotype is conducted within the cohorts excluding individuals with the KIR Bx haplotype and KIR2DS4-negative individuals. *p* < 0.05: statistically significant based on two-tailed Pearson chi-square test or ^A^Fishers’s exact test (two-tailed)

More in-depth, the KIR genotypes can be further divided into two regions, centromeric (Cen) and telomeric (Tel). Based on the gene contents of these regions, Cen and Tel regions can be classified as either an A or B type region (Cooley et al. 2010). Comparing the distribution of the presence of Cen-A and Cen-B or Tel-A and Tel-B regions among MM patients and healthy controls did not reveal an association between the regions and presence of disease (Table 4). Moreover, the number of B genotype-defining genes in these centromeric or telomeric motifs, previously annotated as the KIR B-content score (Cooley et al. 2010), did not differ between patients with MM and healthy controls (*p* = 0.964).

### KIR-HLA ligand pairs and NK cell education potential

We next evaluated the potential association between KIR-HLA ligand pairs and the occurrence of MM. Individuals can either be educated through C1/KIR2DL2/3, C2/KIR2DL1, Bw4/KIR3DL1, or a combination of these pairs. We observed that the occurrence of Bw4 with KIR3DL1 was more frequent in MM patients compared to healthy controls, although this did not reach the level of significance (72.7% vs. 63.1, *p* = 0.05, OR 1.557 (0.999–2.427)) (Table 5).

**Table 5:** KIR-HLA ligand pair distribution among study population

	**Control (*n* = 195)**	**Patient (*n* = 172)**		
	*%*	*%*	*p-value*	*OR*
Potential iKIR education through				
*C1 ~ KIR2DL2/3*	91.3	85.5	0.081	
*C2 ~ KIR2DL1*	53.9	53.5	0.945	
*Bw4 ~ KIR3DL1*	63.1	72.7	0.050	1.557 (0.999–2.427)
*One KIR-HLA pair*	23.6	18.0	0.191	
*Two KIR-HLA pairs*	44.6	52.3	0.140	
*Three KIR-HLA pairs*	31.8	29.7	0.657	
*C1 ~ KIR2DL2/3 only*	21.0	16.9	0.311	
*C2 ~ KIR2DL1 only*	2.1	1.2	0.503	
*Bw4 ~ KIR3DL1 only*	0.5	0.0	1.000^A^	
*C1 ~ KIR2DL2/3* *and C2 ~ KIR2DL1*	13.9	9.3	0.177	
*C1~ KIR2DL2/3* *and Bw4 ~ KIR3DL1*	24.6	29.7	0.278	
*C2 ~ KIR2DL1* *and Bw4 ~ KIR3DL1*	6.2	13.4	0.019	2.453 (1.134–4.888)
*C1 ~ KIR2DL2/3* *and C2 ~ KIR2DL1* *and Bw4 ~ KIR3DL1*	31.8	29.7	0.657	
Potential aKIR education through				
*C1 ~ KIR2DS2*	47.9	44.2	0.472	
*C2 ~ KIR2DS1*	22.6	19.2	0.428	
A*11 ~ KIR2DS4	11.0	11.3	0.943	
*C2 ~ KIR2DS5*	19.5	15.7	0.343	
B*51 ~ KIR3DS1	3.08	6.40	0.131	
Other ***KIR-HLA*** pairs				
A*3 and/or A*11 ~ KIR3DL2	39.0	32.6	0.201	

Comparison of the distribution of interacting KIR-HLA ligand pair in MM patients (*n* = 172) compared to healthy controls (*n* = 195). Education is approximated as the co-occurrence of the HLA NK-epitope and the corresponding KIR in an individual. *p* < 0.05: statistically significant; based on two-tailed Pearson chi-square test or ^A^Fishers’s exact test (two-tailed). *iKIR* inhibitory KIR. *aKIR* activating KIR

When comparing combinations of KIR-HLA ligand pairs, we observed that potential education through two KIR-HLA ligand pairs, C2/KIR2DL1 and Bw4/KIR3DL1, was more likely in MM patients, compared to healthy controls (13.4% vs. 6.2%, *p* = 0.019, OR 2.453 (1.134–4.888) (Table 5). No differences were observed between MM patients and healthy controls for the total number of KIR-HLA ligand pairs.

KIR3DL2 is able to interact with HLA-A*3 and A*11, although this interaction is peptide dependent (Hansasuta et al. 2004). Our analysis did not reveal any significant differences in the frequency of occurrences involving either one or both of HLA-A*3 or HLA-A*11 in conjunction with KIR3DL2 between MM patients and healthy controls (Table 5).

While NK cell education by inhibitory KIRs (iKIRs) translates into effective sensing of missing self HLA class I targets, activating KIR (aKIRs)-mediated education induces hypo-responsiveness and impairs NK cells responsiveness (He and Tian 2017). Although the HLA ligands for all aKIRs have not been fully characterized, certain educating interactions have been identified. For instance, KIR2DS2 reacts with C1-epitopes, both KIR2DS1 and KIR2DS5 interact with C2-epitopes, KIR2DS4 interacts with HLA-A*11, and KIR3DS1 interacts with HLA-B*51 (Bw4 motif) (Carlomagno et al. 2017; Cyril Fauriat et al. 2010; Pende et al. 2019). No significant differences were observed in the frequencies of these KIR-HLA ligand pairs between MM patients and healthy controls (Table 5).

## Discussion

In the present study, we investigated the distribution of KIR genes, the presence of HLA-class I NK-epitopes, and their co-occurrence in MM. Compared to healthy controls, we did not observe specific KIR genes or genotypes, or HLA NK-epitopes with higher prevalence among MM patients. However, MM patients were more likely to be C1^−^/C2^+^/Bw4^+^ and co-occurrence of Bw4 and KIR3DL1 was more common in MM patients. Furthermore, MM patients were more likely to have both the KIR2DL1/C2 and the KIR3DL1/Bw4 NK cell-educating KIR-HLA ligand pairs.

Previous cohort studies have described KIR genes or combinations with altered expression in MM patients compared to healthy controls. KIR2DS5, the allele KIR2DS4*001 (Hoteit et al. 2014), and the combination of KIR2LD1^−^/KIR2DL2^+^/KIR2DL3^−^ (Martínez-Sánchez et al. 2016), have been reported to be significantly more prevalent among MM patients compared to the healthy control population. We did not observe these differences in our study population. These discrepancies might result from the limitations of relatively small cohorts (34 (Hoteit et al. 2014) and 25 (Martínez-Sánchez et al. 2016) MM patients), which could have skewed observations in either study. Additionally, our analysis did not extend to the allele or haplotype level of KIR genotypes. As certain KIR-alleles may have distinct functional properties or affinities for HLA ligands, studying these could help elucidate their functional significance in immune response and disease outcome. Consistent with previous cohort studies in MM assessing KIR AA vs Bx genotypes (Beksac et al. 2023; Hoteit et al. 2014; Theeranawakam et al. 2021), our findings revealed no statistically significant disparities in the distribution of AA vs Bx genotypes.

Our study also examined the presence of HLA NK-epitopes and the co-occurrence of NK cell-educating KIR-HLA ligand pairs. We observed that MM patients were more likely to be C1^−^/C2^+^/Bw4^+^, compared to healthy controls. In a similar fashion, Beksac et al. (2023) identified that MM patients were more likely to be C2^+^ homozygous; however, Bw4 was not included into these analyses. In our study, we observed a higher prevalence of HLA-Bw4 motifs together with KIR3DL1 and an increased prevalence of the two NK cell educating KIR-HLA ligand pairs KIR2DL1/C2 and KIR3DL1/Bw4 among MM patients. In addition, we observed increased presence of certain KIR-HLA ligand pairs, but no altered total number of genetically present KIR-HLA ligand pairs. In contrast to our findings, Martinez-Sanchez et al. (2016) reported a higher frequency of complete absence of KIR2DL1 with C2 and an elevated presence of only a single iKIR-HLA-C ligand pair in patients with MM. Again, the differences between our and previous data may be due to the relatively small cohort sizes.

In contrast to the positive impact of interactions between iKIR and HLA ligands on NK cell education, interactions through aKIR can lead to downregulation of NK cell activity in a process called “unarming” (Fauriat et al. 2010). Our study did not observe significant differences in the genotypic expression of the activating KIRs (KIR2DS1, -2DS2, -2DS4, -2DS5, and -3DS1) or the co-occurrence with the respective HLA molecules between MM patients and healthy controls. It is worth noting that while additional specific HLA allotypes can be ligands for aKIRs, the overall resolution of our genotyping methodology was not sufficient to determine these interactions (Pende et al. 2019). Another layer of depth that shapes the NK cell response is the peptide-specific recognition of HLA molecules by aKIR, which was not covered by the current methodology (Sim et al. 2023). Moreover, both self-peptides as well as peptides derived from viruses and bacteria may affect the affinity of KIR for an HLA molecule (Sim et al. 2023). For example, KIR2DS4, recognition of a peptide derived from a bacterial recombinase A associated with HLA-C*05:01 has been reported (Sim et al. 2019). Finally, while additional types of activating KIRs can be expressed by NK cells, the specific ligands for these receptors are currently unknown. Further investigation of additional interactions between aKIR and HLA ligands could potentially shed light on how MM cells evade NK cell response by inducing a state of hypo-responsiveness through interaction with their cognate HLA molecules.

In addition to the beneficial impact of education, interaction through matched HLA-inhibitory KIR will provide a strong inhibitory signal to educated NK cells, serving as an inhibitory immune checkpoint to control their effector function. We have previously demonstrated that both primary MM and MM cell lines retain expression of classical HLA Class I and HLA-E (Sarkar et al. 2015). Therefore, it will be interesting to expand the scope beyond solely genetic profiling of KIR and HLA, such as functionally assessing whether the direct inhibition through the various possible HLA-KIR interactions, with an emphasis on KIR3DL1/KIR2DL1-educated individuals, results in NK cells with different anti-tumor capacity.

Another reason why protein-level analyses are relevant is the fact that the KIR repertoire and expression level on an individual NK cell will affect the education potential and effector capacity (Brodin et al. 2009a, b). The repertoire, functional capacity of a receptor, and expression level are dependent on various factors. For example, KIR Null-alleles result in the absence of expression of the respective KIR (Falco et al. 2013; Pende et al. 2019). Allelic variations in KIR genes further diversify the functional repertoire by altering the avidity to HLA ligands (Frazier et al. 2013). Infection with cytomegalovirus (Béziat et al. 2013a), KIR gene copy number variation (Béziat et al. 2013b), and polymorphisms in KIR promotor regions (Bruijnesteijn et al. 2020) also directly impact KIR expression level or the number of KIR expressing NK cells. Hence, conducting high-resolution typing paired with protein-level analysis will allow assessment of the independent contribution of KIR-HLA interactions to NK cell education. Murine models have demonstrated that different receptor-ligand interactions have varying impacts on NK cell education, with strong interactions leading to effective education and weak interactions resulting in compromised cells (Brodin et al. 2009a, b; Johansson et al. 2005). Furthermore, in another murine system, NK cell responsiveness was shown to increase with each added self-MHC-specific Ly49 receptor. This resulted in higher cytotoxic potential against a missing-self target by NK cells with two inhibitory receptors versus NK cells expressing only one or none (Joncker et al. 2009). Functional analyses that investigate whether NK cells from C1^−^/C2^+^/Bw4^+^ individuals or KIR3DL1/KIR2DL1-educated NK cells have altered functionality are warranted to elucidate the consequences of these interactions.

While MM patients and healthy controls show no difference in triple-positivity for HLA NK-epitopes, this observation carries positive implications: as the tumor microenvironment induces a hypo-functional state in patient NK cells, the antitumor potential of healthy donor-derived NK cells is being explored (Fauriat et al. 2006; Pazina et al. 2021; Ponzetta et al. 2015). Of interest is the leveraging of missing-self recognition in an allogeneic setting (Ruggeri et al. 2016), such as haploidentical transplantations. Given our findings, haplo-SCT combined with KIR-ligand mismatching remains a viable treatment option for the majority of MM patients.

Within the context of NK cell education and activation of effector function, it is important to consider the role of NKG2A and its interaction with HLA-E, as this interaction also results in NK cell education. Two types of dimorphisms, one in the HLA-B leader peptide, the other in HLA-E itself, have been described to determine HLA-E surface-level expression (Horowitz et al. 2016; Kanevskiy et al. 2019). Even a modest reduction in surface expression level has been shown to already affect education and functional capacity of NK cells (Hallner et al. 2019). NK cells from individuals with the HLA-B dimorphism resulting in higher HLA-E expression had superior degranulation against the HLA-negative K562 cells and KIR ligand-matched AML blasts (Hallner et al. 2019), indicating better-educated NK cells. Whether these polymorphisms correlate to MM susceptibility or disease progression requires further investigation. While our study did not investigate NKG2A or HLA-E, our prior research has demonstrated that the potential limiting effects stemming from both the education status or the direct inhibitory interaction between NKG2A and HLA-E could be overcome through the inclusion of an ADCC-inducing antibody (Mahaweni et al. 2018).

Two potential confounders could have influenced the data. First, various ethnicity-specific HLA and KIR profiles have been described (Sanchez‐Mazas et al. 2011) and we do not have in-depth information on the ethnic background of our cohort. However, the overall distribution of KIR gene frequencies and the AA vs Bx genotypes in our study was comparable to the general Caucasoid population (Cisneros et al. 2020; Manser et al. 2015). Second, MM is predominantly a disease that manifests in later stages of life, indicating a higher risk of its development among older individuals and our control and patient groups were not age matched. In this context, if specific KIR or HLA signatures were found to be correlated with improved aging or increased longevity, it would raise the possibility of a potential association of these KIRs or HLAs with MM incidence. However, while certain HLA-DRB1 and some HLA Class I alleles have been observed to show a positive association with longevity (Ivanova et al. 2019), there are no definitive indications that the presence of certain HLA or KIRs significantly impacts longevity.

In conclusion, our findings demonstrate several associations between specific HLA NK-epitopes and KIR combinations and the occurrence of MM. However, the differences between patients and healthy controls primarily manifested within the less common genotypic variants. As a result, the predictive utility of a less prevalent genotype for disease occurrence in the total patient population may have some constraints. Larger studies or a meta-analysis of previous cohort studies might contribute to stronger correlations. Possibly more important might be functional analyses, for which the observed genetic associations might indicate which patient subgroups should be included in initial further investigations.

## Data Availability

The original contributions presented in this study are included in the article, further inquiries can be directed to the corresponding author.

## Declarations

G.M.J.B. is the Chief Executive Officer/Chief Medical Officer/Co-founder of CiMaas, BV, Maastricht, the Netherlands.
